# Durable Local Control With Preserved Renal Function for Stereotactic Body Radiotherapy in Cryoablation-Refractory Clear Cell Renal Carcinoma

**DOI:** 10.7759/cureus.68864

**Published:** 2024-09-07

**Authors:** Parker Heger, Keaton Rummel, John Watkins

**Affiliations:** 1 Surgery, University of North Dakota School of Medicine and Health Sciences, Grand Forks, USA; 2 Radiation Oncology, Mayo Clinic, Rochester, USA; 3 Radiation Oncology, Bismarck Cancer Center, Bismarck, USA

**Keywords:** progression-free survival, general radiation oncology, renal cell carcinoma (rcc), oligoprogressive, sbrt (stereotactic body radiotherapy)

## Abstract

Renal cell carcinoma (RCC) is the most common type of kidney cancer, accounting for most renal cancers. Oligoprogressive RCC (OP-RCC) describes metastatic RCC wherein one or a few metastatic sites continue to progress, while the majority of metastatic sites are stable on systemic therapy. Treatment options for the primary site for OP-RCC include cytoreductive nephrectomy, stereotactic body radiation therapy (SBRT), or ablative techniques, although there is no currently agreed-upon standard for treatment. This report describes a 76-year-old male with OP-RCC who was treated with salvage SBRT after failing cytoablation therapy. A review of the current literature on SBRT as a treatment option for OP-RCC is presented and discussed. This case demonstrates that SBRT may be a viable salvage treatment option for patients with OP-RCC that provides good local disease control while preserving long-term renal function.

## Introduction

Renal cell carcinoma (RCC) is the most common type of kidney cancer, accounting for roughly 80% of all malignant tumors found in the kidney and 2% of all cancers diagnosed globally [1]. The surveillance, epidemiology, and end results (SEER) statistics program reports that in the US, there will be an estimated 81,800 new cases in 2023 alone [2]. Approximately one-third of cases will be diagnosed as metastatic and of the cases diagnosed as nonmetastatic, 20-50% will progress to metastatic disease. The median survival rate for metastatic RCC is about 13 months, and the five-year survival rate is below 10% [3].

Oligoprogressive-RCC (OP-RCC) refers to a disease where one or a few metastatic lesions continue to progress despite systemic therapy, while most of the metastatic lesions are either stable or show signs of regression [4]. Local therapies to the primary tumor site can be indicated in oligometastatic RCC or OP-RCC settings. Given that the survival rate for metastatic RCC is low, aggressive treatment is often necessary to provide patients with the best chance of survival. Treatment options for the primary site for OP-RCC include cytoreductive nephrectomy, stereotactic body radiation therapy (SBRT), or ablative techniques [5]. In cases of metastatic disease, such as OP-RCC, patients’ options are often limited to metastasectomy, SBRT, enrollment in a clinical trial, first-line systemic therapy, or supportive care. There is currently no agreed-upon dosing and frequency for SBRT in the setting of metastatic RCC [6]. Here, we present a case of a patient with oligoprogressive metastatic RCC who was treated with salvage SBRT after failing cryoablation therapy.

## Case presentation

A 76-year-old male patient presented with chronic low back pain that began seven years prior. His low-back pain was previously responsive to steroid injections but became progressively worse over six months and started to include radicular left leg pain. Lumbar spine MRI demonstrated lesions that are concerning for metastases, including extraosseous extension at the L2, L4, and L5 levels. Additionally, an exophytic right renal mass was also partially visualized; a subsequent CT scan characterized a 6.1 cm exophytic right renal mass, spine and pelvic osseous lesions, a 1.2 cm right adrenal nodule, and a 5 mm right lung nodule. A biopsy of the L4 lesion demonstrated high-grade clear cell carcinoma consistent with a renal primary mass. A brain MRI was negative.

The patient went on to receive palliative radiotherapy in the lumbar spine and right sacroiliac joint with 3,000 cGy in 10 once-daily fractions. Following his course of palliative radiotherapy, pembrolizumab monotherapy was started, and axitinib was added two weeks later.

Following dose adjustments to axitinib because of stomatitis and weight loss over an 11-month period, CT scans demonstrated a new 6-mm right lower lobe lung nodule, stable osseous lesions, and progression of the right renal mass, with indeterminate small liver lesion. The decision was made to change systemic therapy to cabozantinib. Follow-up CT scans four months later showed a decrease in the size of the right renal mass with stability of the osseous lesions. However, the new right lung lesion had grown to 1.6 cm, and there was new enlargement of the thoracic lymph nodes. A biopsy of the mediastinal and hilar lymph nodes confirmed clear cell carcinoma. The decision was made to treat the right lung nodule using SBRT with 57.5 Gy in five fractions coincident with systemic therapy change to nivolumab/ipilimumab (subsequently transitioned to nivolumab monotherapy).

**Figure 1:**
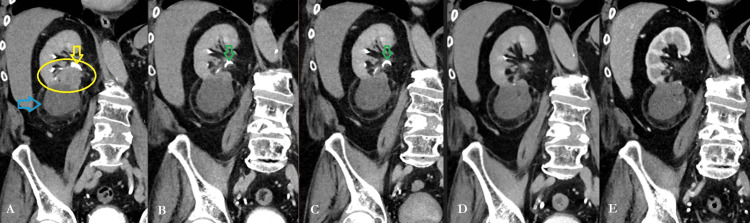
Representative pre- and posttreatment coronal CT images shown at the following intervals: (A) pre-SBRT, (B) four months post-SBRT, (C) six months post-SBRT, (D) 9.5 months post-SBRT, and (E) 15.5 months post-SBRT.

Following SBRT to the right lung nodule and the changes to systemic therapy, serial imaging demonstrated initial stability. However, 11 months following lung SBRT, CT abdomen/pelvis demonstrated significant enlargement of the right renal mass (6.7 cm, previously 4.7 cm). The right renal mass was then treated with cryoablation, while the nivolumab therapy was still ongoing. Seven months post-ablation, CT imaging demonstrated a 1.7 cm-enhancing focus at the superior aspect of the ablation cyst in the anteroinferior right kidney adjacent to the renal pelvis and exiting ureter (Figure 1, Panel A). A subsequent MRI three weeks later confirmed a 4.5 cm heterogeneously enhancing residual/recurrent lesion at this site, with a suspected tumor thrombus in the right renal vein. The disease remained well-controlled at all other sites.

**Figure 2:**
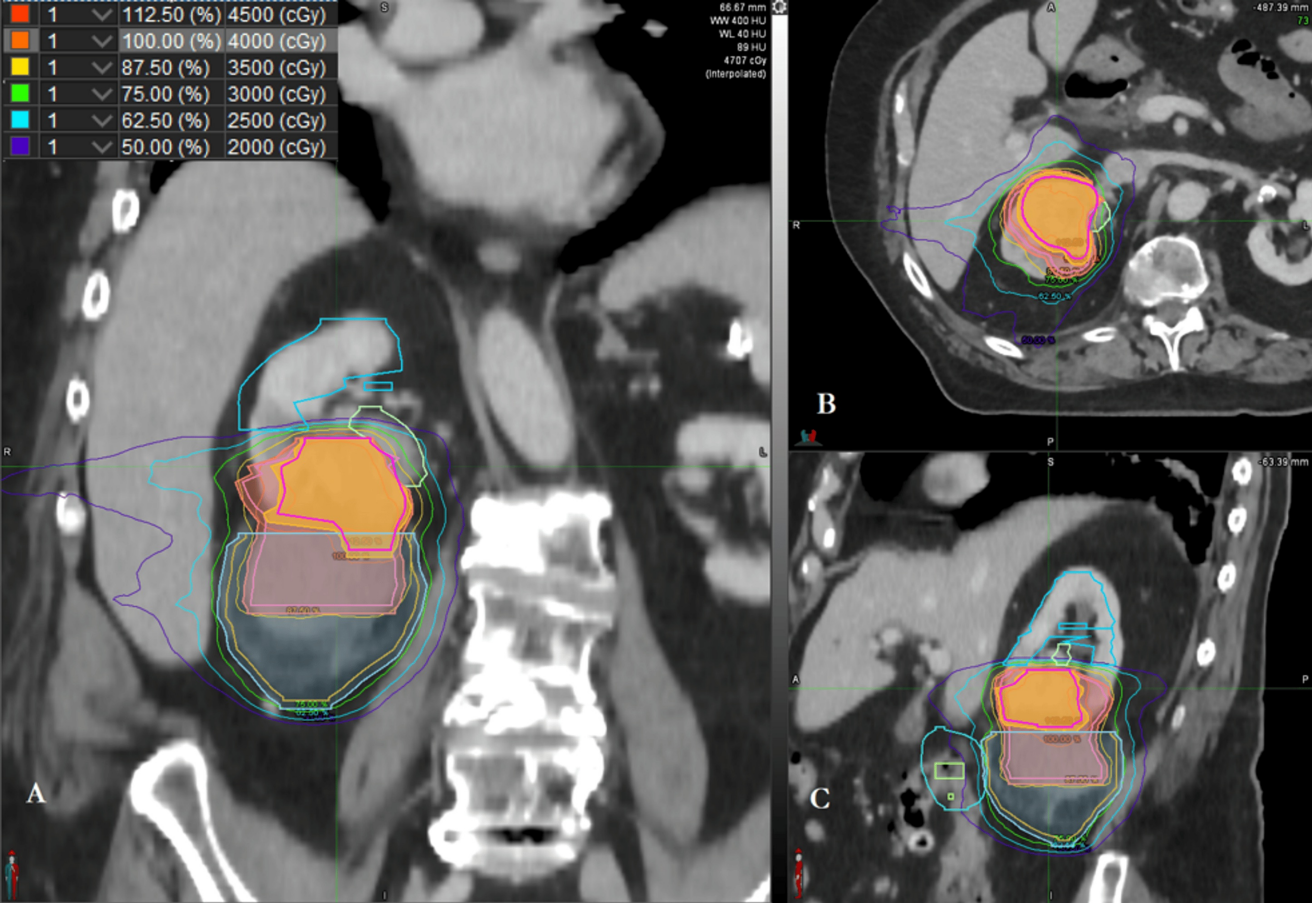
Right renal SBRT isodose maps from treatment planning CT scan shown in the (A) coronal, (B) axial, and (C) sagittal planes.

Following the discussion of the updated imaging and consideration of management options, the patient elected to proceed with definitive SBRT salvage of the right renal cryoablation failure site. The rationale for selecting SBRT included focusing on the progression of the lesion in a patient who was otherwise doing well on systemic therapy. The patient also preferred SBRT to more aggressive treatments such as surgical management given metastatic disease. Technetium-99m-labeled MAG-3 was performed, with differential renal perfusion profile of 49% right and 51% left demonstrated, and incidental mention of paucity of uptake in the inferior right renal pole. A vesicoureteral stent was placed for localization with the intent to leave the stent in place for six months post-salvage SBRT to reduce the risk of subacute/late proximal ureteral stricture. Daily cone beam CT (CBCT) with real-time surface-based image guidance in mid-exhalation breath-hold was used, with 5 mm (transverse) to 8 mm (craniocaudal) planning treatment volume (PTV). An SBRT treatment plan was created using a 6 MV flattening filter-free volume-modulated arc therapy (FFF-VMAT) with 4,000 cGy in five fractions given over nonconsecutive days to the renal tumor. Synchronous doses of 3,500 cGy and 3,000 cGy were prescribed to a 5 mm rim of adjacent renal tissue plus the cryoablation cyst and the entire cryoablation cyst, respectively. Representative isodose map images are demonstrated in Figure 2.

For therapy setup, axial 2 mm slice thickness images were acquired from the dome of the liver through the pelvis, with rigid immobilization and low abdominal belt compression, acquired with both free breathing and respiratory-gated techniques, from which customized internal target volume (ITV) expansions were based. Given the use of pretreatment CBCT, respiratory immobilization, and real-time surface image guidance, PTVs were limited to 2 mm for the highest dose region (4,000 cGy) and 3 mm for intermediate and lower-dose regions (3,500 and 3,000 cGy, respectively).

**Figure 3:**
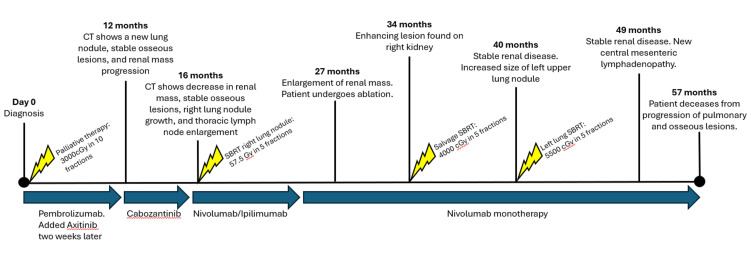
Treatment timeline and disease course.

The patient tolerated the treatment well, experienced no acute adverse events, and had minimally changed renal function (creatinine 1.19-1.63 mg/dL posttreatment; 1.22-1.42 in the six months before salvage SBRT). He has been able to continue with nivolumab monotherapy. At six months following salvage right renal SBRT, a CT demonstrated stable posttreatment changes at the treated site (Figure 1, Panel C), with increased size of peripheral left upper lobe lung nodule (9 mm, abutting chest wall), treated with SBRT (5,500 cGy in five fractions on nonconsecutive days), with favorable tolerance and radiographic response 3.5 months later (local control noted at caudal renal site; Figure 1, Panel D). A dose of 5,500 cGy was employed on the second lung nodule because of the smaller lesion size (9 mm rather than 1.6 cm) and prior thoracic radiotherapy in the context of ongoing immunotherapy to maximize local control (BED >100) while minimizing the risk of pneumonitis. Nine months later, the stability of the treated right renal target was again noted (Figure 1, Panel E); however, new central mesenteric lymphadenopathy was appreciated, consisting of at least four suspicious-appearing soft tissue densities, up to 1.3 cm maximal axial dimension. Nearly two years posttreatment, the patient's creatinine remained stable between 1.26 and 1.50. Unfortunately, the patient deceased at 23 months post-SBRT from pulmonary and osseous metastatic progression. The renal SBRT site remained controlled at the final CT scan, 18 months posttreatment. Figure 3 shows an overview of the patient's disease course and treatment timeline.

## Discussion

Our case demonstrates the utility of SBRT to the renal primary site in oligoprogressive metastatic RCC. In the case of oligoprogressive metastatic RCC, the typical management is to change systemic therapies. However, targeted SBRT to the sites of oligoprogression while continuing the same systemic therapy has been shown to delay the need to change systemic therapy. Studies by Cheung et al. and Hannan et al. demonstrated a median time to change systemic therapy post-SBRT to oligoprogressive sites of 12.6 months and 11.1 months, respectively [5,7]. In our case, the patient has been able to delay changing systemic therapy because of the success of salvaging SBRT to the right renal mass and is now nearly two years post-renal SBRT.

Progressive renal dysfunction can be a concern with renal SBRT. A couple of studies have examined the impact of SBRT on kidney function over time. Our patient has a posttreatment creatinine of 1.19-1.63 mg/dL compared to 1.22-1.42 in the six months before SBRT. Moreover, our patient's creatinine remains stable nearly two years posttreatment. Prior studies have demonstrated that there is a longitudinal decline in renal function following renal SBRT, with one study showing a change in eGFR of -11.5 at two years post-SBRT and of -14.0 at four years post-SBRT [8]. However, the FASTRACK II clinical trial found that patients who had undergone SBRT for primary RCC had acceptable kidney function at 43 months posttreatment, which furthers the evidence that SBRT is a safe treatment option for these patients [9]. While SBRT does have a favorable toxicity profile, it is prudent to continue to monitor renal function following renal SBRT. Fortunately, to date, our patient has experienced minimal change in overall renal function following renal SBRT.

Historically, radiotherapy has had little role in the treatment of RCC. RCC has long been understood to be a radioresistant tumor. However, the ablative doses used in SBRT have been demonstrated to achieve effective local control with a favorable toxicity profile when used in both primary RCC and metastatic RCC [10,11]. A recent systemic review notes that SBRT could be an alternative therapy when treating primary RCC in patients who decline surgery, are medically inoperable, or are technically high risk [12]. Other reasons to consider SBRT in patients with limited treatment options include the preservation of quality of life and renal function, maintaining patient eligibility for clinical trials, and the potential for enhanced systemic response when combined with immunotherapy. This question of whether cytoreductive renal SBRT in addition to immunotherapy for metastatic RCC is beneficial is actively being investigated by the current CYTOSHRINK (NCT04090710) and SAMURAI (NRG-GU012) trials. It stands to reason given the success of SBRT with treating primary RCC and metastatic RCC lesions that SBRT may be a useful cytoreductive treatment to the primary renal mass in the setting of metastatic RCC apart from standard-of-care immunotherapy and imbuement of local control and survival benefits.

## Conclusions

Renal SBRT is an effective local therapy in the setting of OP-RCC. Our case demonstrates that renal SBRT can be used successfully as a salvage treatment for oligoprogressive disease that provides local disease control and preserves long-term renal function. Fortunately, our patient has had minimal overall renal function change, but clinicians should be aware of the possibility of a decline in renal function, and continued monitoring of renal function post-SBRT is warranted.
